# Formulation in eating disorder focused family therapy: why, when and how?

**DOI:** 10.1186/s40337-021-00451-3

**Published:** 2021-08-10

**Authors:** Julian Baudinet, Mima Simic, Ivan Eisler

**Affiliations:** 1 Maudsley Centre for Child and Adolescent Eating Disorders (MCCAED), Maudsley Hospital, De Crespigny Park, Denmark Hill, London, SE8 5AZ UK; 2 Institute of Psychiatry, Psychology and Neuroscience (IoPPN), King’s College London, De Crespigny Park, London, SE5 8AB UK

**Keywords:** Formulation, Maudsley family therapy, FT-AN, FT-BN, Family based treatment, FBT, Anorexia nervosa, Bulimia nervosa, Adolescent

## Abstract

In the broadest sense, formulation, or case conceptualisation, is the process of collaborative sense-making. It is something all clinicians are doing constantly, whether formally and consciously or not. Most clinicians are familiar with the general principals and need to formulate as it is a core part of clinical training across most disciplines. Treatment manuals often discuss formulation, but the practical steps involved and the process of developing, building upon and revising formulations are not always provided. In clinical practice, the active use and continued development of formulation during treatment is not always prioritised, especially beyond the initial assessment. This could be due to a number of reasons, such as a potential lack of clarity, understanding or confidence in the use of formulation across treatment, a lack of dedicated time and reflective space for formulation in the context of increasingly busy clinical settings, or a perception that it may be less necessary in the context of treatment approaches that are more structured and prescriptive. This article outlines the use of formulation in family therapy for child and adolescent eating disorders, provides guidance on how to actively include formulation more throughout treatment and discusses why this might be useful.

**Supplementary Information:**

The online version contains supplementary material available at 10.1186/s40337-021-00451-3.

## Plain English summary

All clinicians, families and young people are constantly trying to make sense of the difficulties people present to treatment with and how it progresses once it has started. This process is called formulation and is occurring whether people are conscious of it or not. It is through the formulation process that a person’s specific difficulties are understood, how treatment goals are established, and how treatments can be more effectively reviewed when there is a lack of progress. Despite these potential benefits, formulation is not always prioritised in day-to-day practice. This article outlines formulation practices in eating disorder focused family therapy. It then provides guidance on why, when and how formulation could be incorporated more consistently. Potential benefits and pitfalls are considered, as well as considerations of how to make sure the formulation is collaboratively developed with the families and team to avoid individual biases and assumptions.

**Supplementary Information:**

The online version contains supplementary material available at 10.1186/s40337-021-00451-3.

## Background

In the broadest sense, formulation is the “process of ongoing collaborative sense-making” (p.4) [1]. All clinicians (and their clients) are constantly formulating, regardless of discipline, treatment model, or how formal and explicit the process is. It is the tool through which clinicians link theory to practice [2] and personalise treatment to the individual [3]. It integrates the person or family’s current experiences with the clinician’s and team’s knowledge of theory, research and clinical experience [4]. Importantly, formulation is not a list of events, behaviours or internal experiences, as might be produced after an assessment. Rather, formulation is the synthesis of this information into a narrative that gives meaning to *how* and *why* these factors co-occur.

Formulation is a core component of most psychotherapies, although it goes by different names (e.g., case conceptualisation, case formulation, hypothesizing). As such, the process can look quite different depending on the treatment model and professional discipline. Regardless of the model, all share some key similarities [5–7]. The process of formulating typically includes at least four core components: a) a co-created summary of the person or family’s current difficulties, b) hypotheses concerning the onset, and factors that may have contributed to the development and/or maintenance of the problems c) an explanation of ‘why now’ and in the current context (social, cultural, political etc.), and d) the meaning, or sense, that the person or family make of their difficulties [4, 8].

The process of formulation helps to clarify hypotheses, guide questioning and treatment planning [9]. It also likely plays an important role in engagement and reducing clinician/team bias [9–11]. Formulating collaboratively with a family is also a way of developing a shared narrative, both within the family and between family and therapist, which is a key aspect of developing a therapeutic alliance in family therapy [12].

Within the multidisciplinary team context, formulation can be a useful way of developing a shared team understanding. By drawing on interdisciplinary skills and knowledge, it can improve consistency in team working and helps to highlight individual, team and treatment assumptions [13–15]. The proposed benefits are many, including improving communication and team working, helping to prioritise issues, overcoming biases, improving therapeutic alliance, and ensuring interventions are meaningful to the individual/family [13, 16]. Uniquely, formulating in groups is thought to help ensure a wide variety of perspectives are considered, which ameliorates against individual interpretive errors [16].

While the importance and benefit of formulation are often espoused, it can take a backseat in day-to-day clinical practice. This is likely due to several factors. At times, the process can feel overly complex, difficult to define and hard to document [17]. It is also easy to assume that more complex presentations require complex formulations. This, however, is not always the case and formulation may unnecessarily complicate treatment [3], or even distract from the implementation of evidence based practices [18]. Different people may also formulate in very different ways, which raises issues around the validity and reliability of formulation. Furthermore, developing formulations in multidisciplinary teams can also pose challenges [19, 20] as bringing multiple different theoretical models, training and individual levels of experience together can be tricky to synthesise meaningfully.

These issues are only exacerbated by the dearth of empirical investigation into formulation and whether it impacts treatment experience and outcomes. From the data available, the value of formulation is difficult to determine. Teams and clients alike can find it both helpful and unhelpful [13, 16]. Studies that compare individualised versus generic treatment outcomes typically do not find a difference [21–23].

More recently, the focus has shifted towards the formulation process and validity, rather than its presence alone [24, 25]. Allen and colleagues (2016) found that the use of written formulation in one type of adult eating disorder treatment was not, itself, associated with improved outcomes. Rather, the use of a respectful and reflective tone in the formulation predicted improvements in eating disorder symptom severity, and attention to how anorexia developed was associated with treatment acceptability [26]. This highlights that the manner in which formulation is collaboratively developed might help ensure it is useful, instead of overwhelming or unnecessarily complicated.

In order to address some of these issues, Kuyken [27] has proposed five essential criteria for formulation to be evidence-based. First, it needs to be driven by evidence-informed theory. It also needs to be reliable (different individuals agree on it) and valid (can be triangulated with other data such as the client’s experience, outcome measures, or colleagues’ impressions). Importantly, it also needs to improve treatment and outcomes, and must be acceptable and useful. Kuyken [27] proposes that meeting all criteria when formulating will ensure it is theoretically sound, as well as useful and impactful for treatment.

This article aims to outline current formulation practices in eating disorder focused family therapy for children and adolescents. This is followed by a discussion around when and why formulation might be useful. Lastly, a potential way to formulate within FT-ED is outlined.

## Current formulation practices in eating disorder focused family therapy

The umbrella term, eating disorder focused family therapy (FT-ED), encompasses several, similar forms of eating disorder focused family therapies; including Maudsley family therapy for anorexia nervosa (FT-AN) and bulimia nervosa (FT-BN) [28], Family Based Treatment for anorexia nervosa (FBT-AN) [29] and bulimia nervosa (FBT-BN) [30], and parent focused therapy (PFT) [31]. All FT-ED manuals describe a similar treatment model. All are phased, emphasise working with the family rather than *treating* the family, the importance of the parents having a central role managing the child’s eating early on in treatment, and broadening the scope of treatment later once healthier food and eating practices are established and physical health has improved.

Formulation is included in the current FT-AN/BN and FBT-AN/BN manuals, although the focus in each is slightly different. The Maudsley manual describes a process that starts at assessment and early stages of treatment that should be developed collaboratively with the family [28]. The aim of developing the formulation includes introducing new perspective on the illness that are framed in a non-blaming way, and placing emphasis on positive motivation to help, on strengths, resources and hopes for change. Within the FBT-AN/BN manuals, case formulation and illustrations are provided, although the focus is less on individual experience and more on different family structures (e.g., single parent families, presence/absence of siblings etc) and the availability of family resources to implement treatment. Examples and guidance on how to modify treatment for different family structures are also provided [29]. Little attention is given in all current manuals on how to practically and collaboratively formulate with families.

## Why focus more on formulation?

Focusing more on formulation does not necessarily mean treatment will look particularly different for the majority of families. Treatment tasks and phases will remain the same. What may change, however, is the *way* treatment is delivered and the *experience* of treatment. It is through the formulation that treatment is adjusted to target maintenance factors specific to each family. For those who have a poor or mixed outcome, or baseline factors associated with poorer outcomes, formulation may also help clinicians and families to think more clearly together about why things feel stuck and how to more effectively and quickly overcome identified barriers. Importantly, formulation does not mean the causes of the eating disorder need to be discussed or ‘discovered’. Rather the process helps families and clinicians explore the meaning of what is happening.

Butler [9] outlined eight benefits that all apply to FT-ED, including:

- Prioritizing which issues to focus on
- Increase understanding (for YP, family, clinician, team)
- Clarifying hypotheses and questions
- Planning treatment strategies and selecting specific interventions
- Predicting responses to strategies & interventions
- Overcoming bias
- Thinking about (lack of) progress
- Determining criteria for successful outcome

With regard to FT-ED specifically, the main potential benefits include:

1. *To be more specific and targeted to individual family needs*

By definition, an increased focus on formulation will make treatment more individually tailored and targeted to each family’s specific needs, culture and circumstances. Findings from a recent systematic synthesis of qualitative data on families’ experience of FT-ED indicate that some people feel that both individual and family issues can be neglected in current FT-ED practices [32]. Several studies reported that factors contributing to the development of the eating difficulties can unhelpfully be left unexplored [33, 34] and that more therapeutic work beyond eating symptoms is needed for some [35]. Through the process of ongoing, collaborative formulation these issues might be anticipated, identified more readily and addressed sensitively.

2. *To improve engagement, therapeutic alliance and containment*

By being more targeted and specific, formulation could also help improve engagement, therapeutic alliance and containment for the family. Connecting what has happened for a family to what needs to happen moving forward, can help individuals and families feel more understood. Qualitative examination of the process of change during FT-ED highlights the importance of ‘relational containment’ as a potential treatment mechanism [36]. This is a process whereby the therapist and their team contains the family through the treatment structure, therapist stance and setting, which in turn helps parents to be more effective and contain the young person [36]. Collaborative formulation has the potential to add to relational containment by ensuring families feel included in decision making throughout treatment. Therapeutic alliance is considered a key component of any psychological therapy and has been linked with improved outcomes in FT-ED [37, 38]. Specifically, families who build a shared sense of purpose during treatment have been shown to have improved outcomes [38].

Within family therapy, therapeutic alliance occurs with each individual, with the family as a whole, and between family members. The degree to which families agree on the nature of the problem and the value placed on participating in treatment varies. A recent meta-analysis showed that greater alliance is significantly associated with improved outcomes. Furthermore, the more split, or unbalanced, the alliance is within families, the poorer the outcomes [12]. By being more formulation driven in FT-ED, there is potential for everyone to feel better understood, which could help unite the family around the tasks required in treatment. It is from this foundation, that therapeutic techniques can most effectively be adapted to each unique family’s needs [39].

3. *To strengthen the review process when treatment is not progressing*

By ensuring FT-ED is individually targeted and sensitive, an increased focus on formulation may also help to engage and improve outcomes for those who have a poor response. A recent qualitative study exploring parental experiences of FBT after poor or partial response highlighted that parents felt treatment inadequately addressed their feelings of guilt and blame, that raising anxiety and allocating refeeding responsibility to the parent was not always helpful, and that a behavioural focus came at the cost of not incorporating wider difficulties into treatment [40].

One commonly stated benefit of team formulation is an increase in individual and team understanding, and a reduction in individual/team bias [8, 13]. By being more explicit in the collaborative formulation process throughout treatment, clinicians, families and teams may be better able to make more informed decisions around when and how much to use specific treatment components more sensitively. This includes managing high or low anxiety effectively, negotiating the responsibility for the task of refeeding and how to explore feelings of guilt.

4. *To promote reflective functioning*

Relatedly, formulation may help promote reflective functioning. Focusing on the meaning of events and behaviours as experienced by different family members, and by explicitly linking past and present family and individual experiences, the complexity of stuck points will likely be more fully understood. Promoting perspective taking is a core part of relational connection and attachment [41], which helps to improve understanding and validation within families. Excessive certainty about others’ mental states has been shown to be predictive of poor FT-ED outcomes [38], suggesting all attempts to promote reflective capacities are potentially useful.

5. *To include family and clinician experiences into treatment planning and delivery*

From the clinician’s perspective, several studies indicate a reluctance to adopt a “one-size-fits-all” approach to FT-ED [42, 43]. Some clinicians have said FT-ED can feel overly rigid and focused on behavioural change, to the detriment of adolescent and family engagement and empowerment [42]. This tension around whether to prioritise adherence to evidence-based manuals or to follow clinical experience is not unique to FT-ED, nor is a full discussion of this issue within the scope of this paper. However, formulation is one way of effectively integrating both these elements into treatment delivery. It has been argued that formulation is at the heart of evidence-based practice as it is the tool via which individual experience is combined with theory and evidence [44]. This does not mean that treatment plans or protocols need to be abandoned at the first sign of difficulty. Rather, it allows for family and clinician experience to be considered and to influence the way treatment is delivered.

## Developing a preliminary formulation

Formulation is not an attempt to provide an aetiological account or causal explanation of why a particular individual developed an eating disorder. It aims to offer a meaningful narrative to the young person, their family and clinician of the complex information under the headings described below. The formulation narrative has to be close enough to the family’s own understanding and should incorporate the different perspectives and perceptions that family members may have. At the same time the formulation also has to offer some new understandings from the way that the therapist and the MDT have integrated the different information. The language used in developing the formulation is key. It should be non-blaming and respectful of all family members allowing each family member to feel that their individual perspective and experiences have been understood. When describing behaviours, it is important to differentiate between what was intended by the behaviour and how the behaviour was experienced. A step-by-step guide is presented below and an example formulation provided in the supplementary material (see Additional file 1).

### Step-by-step guide

The process of developing a formulation starts at the beginning of treatment drawing on relevant referral information, as well as observations and information from the assessment. This then serves as the foundation for the ongoing process of formulation during treatment. These steps are likely familiar to most clinicians from any discipline and draw from several different models.

**Fig. 1:**
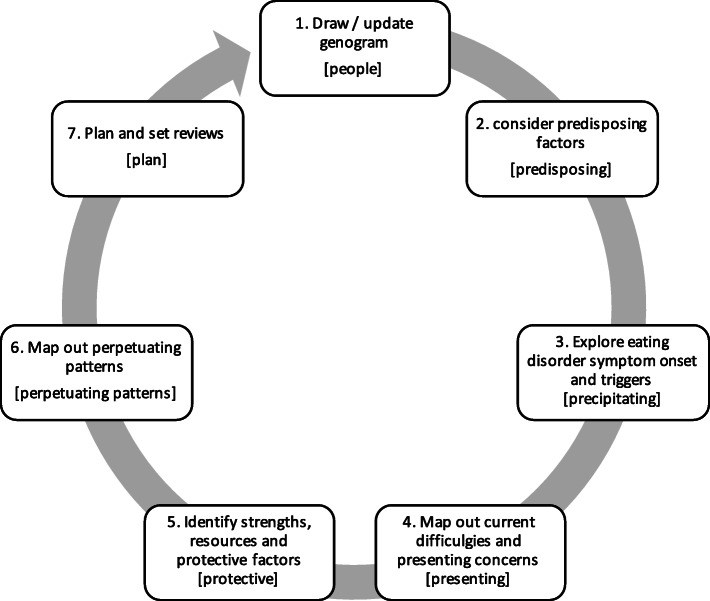
Step-by-step guide to FT-ED formulation

The proposed process can be thought of as a modification and extension of the ‘5 P’s’ [45] to a ‘7 P’s’ formulation (see Fig. 1). It adds two new P’s (people, plans) to the existing 5 P’s (presenting, predisposing, precipitating, perpetuating, protective factors). The context in which all the factors are positioned is added to each, and perpetuating factors are more specifically identified and explored as perpetuating patterns. We emphasise this is just one way to formulate and encourage adaptations and creativity to match your own context. Clinicians can also spend as much or as little time as needed on each step, although all are important to consider briefly. One benefit of this model is that it is integrative and can combine multiple theoretical perspectives.

During each step, it is encouraged to consider what is happening *and* the context/situation in which it occurs. Clinicians should also consider how descriptions are likely to be perceived by each family member.

#### Genogram and network of important relationships

The FT-ED formulation process begins with drawing a genogram, or family tree. Three generations can be useful to ensure parental family of origin stories are included into the treatment process early. This serves as an initial map of current relationship patterns within the family, which will also help with identifying relational resources. It is also a reminder of the importance of including the different perspectives and different understandings that each family member might have.

#### Predisposing factors – anxious temperament, high threat sensitivity, perfectionism, personality traits

Consider personality and bio-temperamental factors for different family members and how this may influence current behaviours and patterns. Distinct bio-temperamental and neurobiological factors have been associated with different eating disorders and malnutrition [46, 47]. Understanding these will ensure treatment is best matched to the individual. It can also be very validating for the individual and family. For adolescents with anorexia nervosa, the task of refeeding for someone who is highly rigid, perfectionistic and threat sensitive, will look considerably different to someone who is more impulsive and emotional labile, temperamentally. Similarly, parental style (e.g., strict, rule-governed, laissez-faire, hands-off) can be considered at this step and how this is experienced by the young person.

##### Consider context

The child’s experiences are partly shaped by family responses. These responses will, in turn, be influenced by broader cultural narratives about gender, race, etc. For instance, an anxious temperament may be viewed as sensitive in girls or cowardly in boys. Genes and family environment interact in variable ways in different families. For example, an anxious temperament will be expressed differently if:

- Shared with one or both parents
- Accommodated by parents (may contain but maintain anxiety)
- Parent(s) themselves overwhelmed by anxiety (may increase anxiety)
- Dynamic between parents around anxiety also impacts parental response

*(Example questions: Does your child worry a lot? Are they a perfectionist? Is anyone else in your family a worrier or perfectionist? How do you, as parents/family members, respond when they seem preoccupied with worry? Does everyone respond in the same way? In what ways do people’s responses differ?)*

#### Precipitating factors

Life events and family vulnerability factors can help to better understand the context in which the illness developed. Some more common factors include low self-esteem, high stress, bullying, loneliness, a sense of not belonging to a group or community. These can all trigger disordered eating or food restriction. In a person with low self-esteem and confidence an innocuous comment can trigger food restriction.

Young people and families may describe events that might seem objectively unimpactful but were very meaningful to the adolescent or family (e.g., a parent changing jobs, family illness, transition from primary to secondary school, or from one school year to another). Occasionally, changes in eating and consequential weight loss are initially triggered by physical illness. It is important to be clear that a trigger for changes in eating is not “the cause” of the eating disorder. It is a factor associated with a change. This will help avoid blame and guilt.

##### Consider context

Include beliefs, intentions and emotional response from the young person, family and close others to possible precipitating factors. Consider how beliefs, norms and emotional response interact to reinforce a particular interpretation of the events. For example, if a young person’s weight loss was precipitated by moving schools, different family members may have different beliefs about this. Parents may feel guilt for putting pressure on their child to choose one school over another, yet may also feel frustrated that their child is struggling with something their siblings found manageable. Similarly, the young person may feel angry at the parents for insisting on the move, yet ashamed for struggling so much with something that others in the family have coped with. Bringing this understanding to the formulation may help the family understand what drives frustration or inconsistencies in certain behaviours. (*Example questions: What else was happening around that time – for you, for the family, in your lives/community more generally? How did family member cope with the change in life circumstances? Was there a difference how people coped? Were those differences spoken about? What impact did the change in circumstance have on the family as a whole and/or each individual family member?)*

#### Presenting concerns: food restriction, binges, purging, ED cognitions, anxiety, low mood, rigidity

Explore the current main concerns for the family. Different family members may have different concerns, different perceptions of these concerns, and different ways of describing them. An important aspect of exploring a presenting problem is to find possible links between different family members’ perceptions and identify common concerns. The purpose of finding common ground is to strengthen within-family alliance and develop a shared family narrative about common treatment goals.

After understanding the current main concerns, the next step is to develop a more longitudinal understanding of the symptoms. This includes exploration around when and how the symptom presentation changed, and how these related to precipitating and predisposing factors already identified. It is worth keeping in mind that many of the predisposing factors are exacerbated by starvation and malnutrition.

##### Consider context

- Family attitudes and response: the family response impacts the emerging illness and the family in turn is impacted by the illness. Consider family attitudes towards food and eating, as well as family coping styles as these may influence the presenting problem.
- Social context: affiliation and connection with peer groups, and broader cultural norms around healthy eating and eating disorders will influence severity and nature of the presenting problems.
- Family members may have different perceptions/understanding of the nature of the problem. These differences may or may not be clearly expressed.
- The family response will also often be experienced differently by family members (e.g., parental anxiety and attempts to help may be experienced as intrusive and controlling by the young person).

*(Example questions: Who in the family first noticed what was happening? How did different people respond to what was happening? Is everyone concerned about the same thing(s)? Are these differences something that get talked about?)*

#### Protective factors

Protective and resilience factors shape the family’s response to the developing illness. Explore when identified difficulties may not occur, or not as strongly, and why. This can include situations, events, as well as people, activities, hobbies, strategies etc. Focusing on strengths is an often-overlooked step in developing a formulations that is important to building resilience and hope [48, 49]. Strengths are identified two-fold, as current and historical strengths.

##### Consider context

Identify close peer and other relationships and potential support both for the young person and other family members**.** Include previous experience of managing difficult experiences or other vulnerabilities to highlight resilience and potential for learning from difficult life-events. (*Example questions: What’s it like knowing your parents will be there for you no matter what? What did you learn as a family from having to manage [insert event] together? What can you take from that experience that might help you with the current challenges?)*

#### Perpetuating patterns

Perpetuating patterns are emotional and behavioural responses that occur in interactions between people. This can occur between young person and parents, other family members, peer group or treating team that maintain the presenting problem (see also the perpetuating pattern under the plan and review heading)**.** Disabling narratives, family beliefs (shared or unshared), cognitions, and emotion can all contribute to the perpetuating patterns. Narratives include personal and shared beliefs that label behaviours as helpful/unhelpful for recovery, or helpful/unhelpful for the young person’s wellbeing. For example, a parent may believe that if their child becomes very distressed when they insist they need to eat, that they are uncaring parents.

Beliefs that individual family members have about themselves and others will vary, with some being more clearly and visibly expressed and others being less obvious. For instance, an uninvolved parent may be perceived as less caring while at the same time the lack of involvement may also be an expression of helplessness or a fear of getting things wrong and making things worse.

**Fig. 2:**
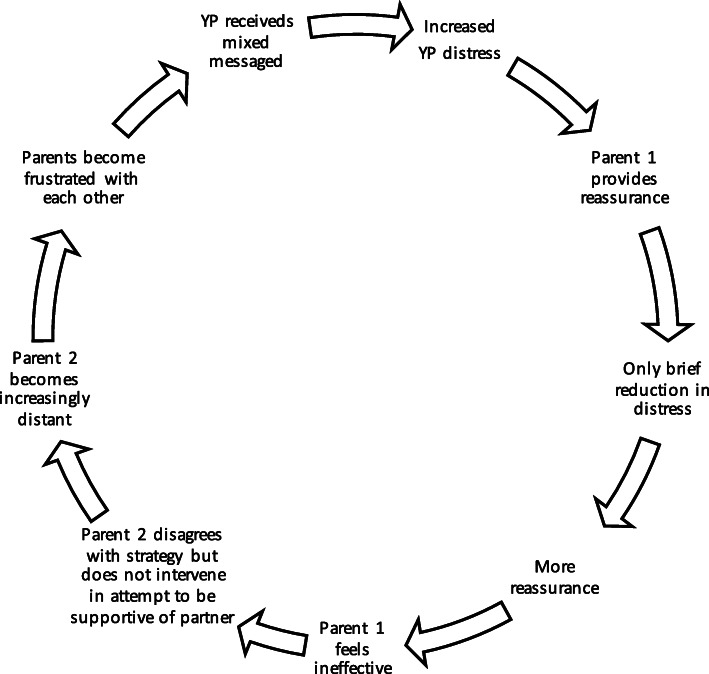
Example of common perpetuating pattern surrounding distress. Abbreviations: YP, young person

**Fig. 3:**
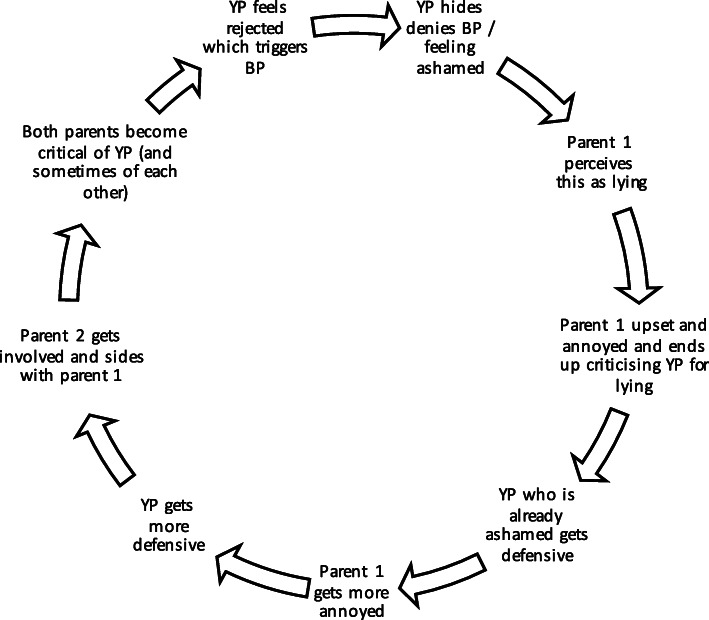
Example of common perpetuating pattern surrounding binge/purge behaviours. Abbreviations: BP, binge/purge behaviour; YP, young person

Any pattern of behaviour can be described in many different ways. They will also be experienced differently by different family members. Patterns are usually circular in nature and include two or more people. The process of drawing patterns is typically completed with the family and repeated for different concerns or behaviours. It is often helpful to emphasise while exploring/outlining/drawing patterns with families that every arrow on the diagram is an opportunity to also explore how to exit the cycle. See below for examples of perpetuation pattern often encountered in clinical practice (Figs. 2 and 3).

When describing perpetuating patterns, the choice of language is of particular importance as these can easily be experienced as attributing blame. The description should be as neutral as possible, differentiate between behaviours, feelings and personality attributes. It needs to utilise language that reflects conversations the therapist has had with the family. This ensures that nothing in the formulation will come as a complete surprise to the family. It should also take into account that the intention behind a behaviour may be experienced quite differently by different people. For instance, consider the difference between describing a person as angry, describing their behaviour as angry, or saying that a person (e.g., child) experiences a comment from another person (e.g., parent) as angry – each of these will be perceived very differently by family members. How much of the perpetuating pattern can be addressed early in therapy will depend on how well the alliance with each family remember has developed.

##### Consider context

Consider the history and development of perpetuating patterns. Many such patterns start with well-intentioned and often sensible behaviours. How a pattern becomes stuck will be determined by a variety of factors. This may include misattribution of intent, or misinterpretation of feelings behind the behaviour, not recognizing the different impact of one’s behaviour in changing circumstances (e.g., age of child), or unexpected life events. Also, reflect on perpetuating patterns within a wider context. This might include social media use, peer difficulties, perfectionistic standards, high expectations around sporting and/or academic achievement, engagement with pro-eating disorder material. (*Example questions include: When [friend/family member] said/did that, what impact did that have on you? How might [insert thoughts/beliefs/ideas] get in the way of moving things forward? What’s it like not being able to agree on this? How might that impact your ability to come together to support each other when things get hard?)*

#### Plan and reviews including relationship with the treating team

The final step is to collaboratively plan treatment targets for the next part of treatment and to set a review point. This ensures treatment is focused, relevant and specific to the family. It will also help clinicians to stay adherent to treatment targets. These can include specific behaviours, relational patterns or emotions that are agreed to be most important at that time. It is often useful to have both eating disorder related goals as well as relational and broader life goals. After assessment, the next review point may be after one-month, given increasing evidence that early change is important for end of treatment outcomes [50–52].

##### Consider team context

**Fig. 4:**
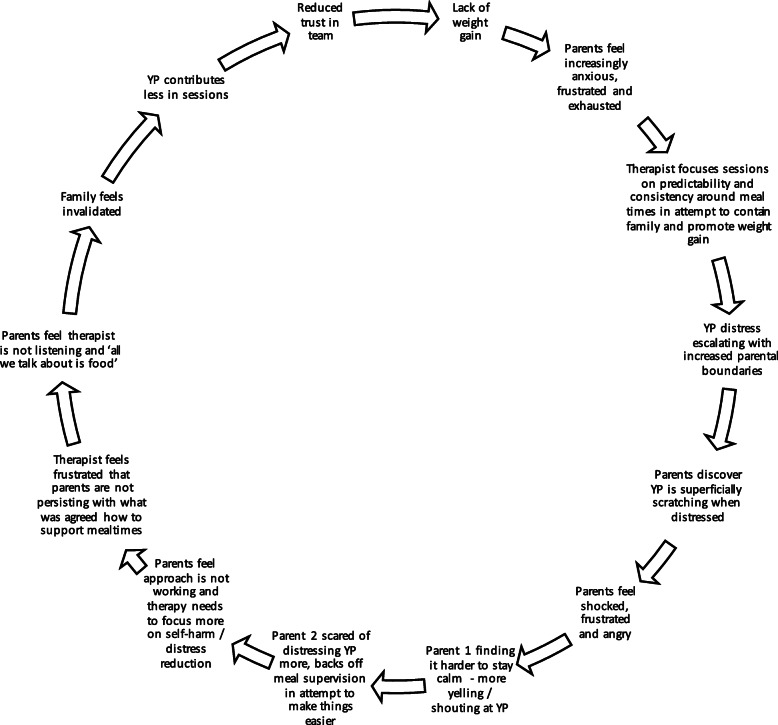
Example of perpetuating pattern that includes team factors. Abbreviations: YP, young person

Regardless of the time point, including team factors into the review process can be useful. This helps expose clinician and team bias as well as how they may be part of a perpetuating pattern. Team factors, as much as individual and family factors, may play a big part in stopping treatment progressing. For example, clinicians who remain very directive in their stance, especially in the latter phases of treatment, may unintentionally disempower families. A formulation-driven review might help identify and shift this. See Fig. 4 for an example of a pattern that includes team/clinician factors (or responses) that can maintain a perpetuating pattern and contribute to the lack of treatment response.

## When and how to review the formulation

The most common time points to review the formulation are listed in Table 1. As a minimum, two time points are suggested: 1) at assessment and 2) at the point of transitioning from focusing on food and eating to broader adolescent and family issues (phase two to three transition in FT-AN/BN). The third most common timepoint is if and when treatment is not progressing. At each of these times, briefly touching upon each of the seven steps can be useful, although there may be no update to some steps.

**Table 1:** Timing and focus of common review points when formulation may be useful

***Timepoint***	***Focus of formulation review***
Lack of weight gain after one month	Understand barriers to early behaviour change and identify further supports
Transition from symptom management to adolescent autonomy	Reformulate difficulties in context of new treatment goals and phase
Sudden deterioration of symptoms	Understand *why* change has occurred *at that particular point of time*
Persistent, high levels of distress	Better understand triggers, meaning and function of distress
Risk event / increased risk (physical health, suicidal ideation, NSSI)	Identify function of behaviour, potential reinforcements of unhelpful coping and ensure safety.
Poor engagement / engagement ruptures	Pause to reconnect and validate to ensure working all together as a team
Endings	Reflect on change points to understand process of change during treatment, strengths and relapse prevention

*Abbreviations*: *NSSI* non-suicidal self-injury

### Revisiting the formulation if treatment is shifting focus

Reviewing the formulation when treatment is shifting focus (e.g., phase two to three transition in FT-AN) allows the clinician and family to review progress to date, confirm new helpful patterns that have emerged, and explore why these have been helpful. By understanding this process and linking it to the formulation, and team factors, the next phases of treatment can be more targeted to the family and nuanced.

If there are no obvious predisposing, precipitating or perpetuating patterns/factors that have hindered treatment progress, this review can focus on new learning during treatment, strengths and new family patterns that have developed that will help with the task of returning to regular life. For others, however, this review may reveal remaining, long-standing issues, such as a high trait anxiety, interpersonal difficulties, bio-temperamental factors such as emotional overcontrol or impulsivity, family difficulties, and so on. A formal review using the formulation helps to explicitly link past, current or future difficulties, which may clarify the most important direction of future work.

Using the example of a young person with a history of bullying and high cognitive rigidity, reviewing the formulation at this stage might help the young person and family more clearly identify what helps the young person to experiment with flexibility, what new skills are needed to manage uncertainty, and social supports needed to ensure food and eating continues independently.

Finally, revisiting the formulation may also be a useful way of ending treatment and marking progress. Reflecting on fully or partially resolved concerns and patterns can emphasise progress.

### Revisiting the formulation if treatment is stuck

The remaining time points outlined in Table 1 are all associated with increased risk or lack of treatment progress. Regardless of the reason, sudden changes or lack of progress are good times to pause and revisit the formulation. New information can be added to all steps outlined above, with the perpetuating patters (step six) often a useful, practical focal point. That way exit strategies can be a focus and provide hope. Patterns around perceived criticism, invalidation, distress or secrecy/hiding, are commonly identified. By mapping these out and including all relevant family and team members/clinician (not just the young person and/or one parent) can help promote understanding, trust and engagement.

### Formulation as a process during treatment

For the majority, the formulation rarely needs to be formally revisited. It can be something discussed in supervision or in team meetings, but not necessarily reviewed formally with the family. For the most part this may be enough, and more explicit attention is not needed. This is particularly true for those who are responding well to FT-ED. Frequently reviewing the formulation may unnecessarily overcomplicate treatment.

However, this does not mean the process of formulating has stopped, nor that it is not collaborative. As aforementioned, it is occurring constantly, regardless of how formal or conscious it is. The task as a clinician is to try and hold the formulation in mind and to update it as treatment progresses, via conversations with the family. This can be a powerful way of validating the young person and family’s experience. For example, a young person’s reluctance to start eating at school may be more linked to past difficult social experiences of having meals with peers, rather than consuming the meal itself. Linking this together can be a useful, formulation driven, way of more effectively problem solving each step in treatment.

## Formulating within a multidisciplinary team

Within child and adolescent eating disorder services, team formulation is likely not needed for every family. It is most useful when a young person has just entered the service, when there is a lack of progress, when treatment is being changed or intensified, and for families with unique presentations. In all these circumstances formulating as a team will help support the family and clinician working with them to take a broader perspective of treatment and make the most informed decision about next steps.

Most teams typically have some type of weekly team case discussion/review. Dedicating some time to review each of the steps outlined above may help to clarify thinking, promote new perspectives for the individual clinician working with the family, and guide decision making. It will also help keep the clinician stay on track with treatment. Formulation together may follow a different format, however, the process of considering each of the seven domains in Fig. 1 remains the same. This can also be done ‘live’ in a reflective team format [53, 54], resources permitting.

Formulating with a reflecting team can also be a useful way for the team to consider more general treatment and team issues. For instance, a team review with a family and clinician who are stuck with ending can act as a useful reminder for all team members involved in the review to reflect on whether their own families are being discharged in a timely manner and what message the team may be unintentionally sending to the family by keeping them in treatment.

## Documenting the formulation

One identified barrier to formulation is that it can be hard to document [17]. As a minimum, clinicians are encouraged to verbally discuss the formulation at the end of the assessment, and to document these seven steps briefly in the file. This can be a very brief document, using bullet-points, diagrams and arrows to link things. It does not need to be neat. For those who progress well in treatment, the formulation document is rarely formally revisited aside from newly identified patterns.

For those families who do not have a linear progression through treatment (which are many), clinicians are encouraged to review and update this document as needed. This can be done in session (to ensure it is collaborative), supervision and case discussion meetings. Mapping patterns is often a focus of these mini reviews, as they become more apparent across treatment. Identifying patters together with the family can be a useful, non-threatening, way of promoting reflection, validation and plan interventions. Some clinicians prefer to review the formulation verbally without separate documentation to standard clinical notes. While sufficient, this has the potential downside of this information being lost in case files and forgotten.

## Potential pitfalls

There are several potential pitfalls of formulation in FT-ED. These include:

1. *Missing context at each step*: Keeping context in mind is important to ensuring the formulation does not become blaming, too focused on one person or one relationship, or an attempt at finding discrete causal factors. By always considering context, including individual difference in responses, intentions, beliefs and perceptions, the formulation Is more likely to remain non-blaming, useful and inclusive.
2. *Using vague language*: This can make the formulation difficult to understand and non-specific. All of which makes it less practically useful to both the family and clinician.
3. *Using language that is (implicitly or explicitly) blaming.* One reason why sharing formulations with families is important is because it requires clinicians to imagine how each family member will experience it. To ensure it is sensitively discussed, clinicians need to use respectful and non-blaming language, that the family themselves uses. This will, in turn, will shape the clinician’s and team’s perception of the family.
4. *Deprioritizing collaboration with the family*: It can be common to discuss and develop formulation with colleagues in supervision or team meetings without including and amending it with the family. This runs the risk of the formulation being less meaningful and potentially invalidating to the family, more open to clinician bias and less useful for setting treatment targets,
5. *The formulation not guiding treatment*: The formulation is only useful if it guides treatment. It is not useful in and of itself. Bringing formulation into reviews and goal setting is the practical way of joining formulation to treatment.
6. *Too detailed or overly complex*: Clinicians might find it easy and interesting to start linking all individual and family events, beliefs and stories. However, if formulation is overly detailed and complex, it can become cumbersome and difficult to use. It may also become overwhelming for the family. Keeping the formulation brief, with simple and respectful language, and linked to treatment goals makes it practically useful. For individuals or families with more complex presentations, focusing on one presenting problem, or one perpetuating pattern at a time, that is collaboratively agreed upon is a way of keeping the formulation and treatment focused on what is most relevant.
7. *Descriptions without meaning*: Without meaningfully integrating different elements, the formulation can become a list of events or behaviours. This will provide more of an assessment summary rather than an understanding of what is happening, or relevant, for the family. It can also make it more difficult to identify meaningful treatment targets.
8. *Too many or too few perspectives:* If too many perspectives on what should be included in the formulation are elicited it can be difficult to construct a meaningful and workable formulation that the clinician can share with the family. Conversely, the opposite can also occur where the formulation is “forcefully trimmed down” to fit the team or clinician’s dominant therapeutic model. Both are especially important to consider when formulating within teams.

## Conclusions

This paper aimed to review current formulation practices within FT-ED, outline the potential benefits and pitfalls, and propose a way in which it could be more readily incorporated into routine clinical practice. We propose that formulation is the basis on which treatment can be adjusted to each family and their specific needs, culture, and circumstances. Nevertheless, it is emphasised that if unnecessarily laboured, it has the potential to block or overcomplicate treatment. Formulation is proposed as a tool to keep in mind throughout treatment. Something that only needs explicit attention if something is not working or the focus of treatment needs changing. At these times, it can be a powerful way of thinking more clearly together, linking current progress to past events and overcoming bias.

### Future directions

The specific benefits of emphasising formulation more in FT-ED is yet to be empirically tested. At this stage, it is an idea being proposed. Investigating whether formulation can improve treatment experience and the review process, especially for those with poorer treatment response, is an important next step. This will likely need to go hand-in-hand with the development of more robust theory outlining how change occurs in FT-ED. The theory of change in FT-ED has been given relatively little focus compared to other psychological treatments. This is not to say that causal factors need to be understood for treatment to be effective, rather a greater understanding is needed of how change occurs during treatment, for whom this process is effective and why.

## Supplementary Information

**Additional file 1.**

## Data Availability

Not applicable.

## Abbreviations

BP: Binge/purge behaviour
FBT: Family based treatment
FBT-AN: Family based treatment for anorexia nervosa
FBT-BN: Family based treatment for bulimia nervosa
FT-ED: Eating disorder focused family therapy
FT-AN: Family therapy for anorexia nervosa
FT-BN: Family therapy for bulimia nervosa
NSSI: Non-suicidal self-injury
PFT: Parent focused therapy
YP: Young person
