# Supplementary material for: Formulation in eating disorder focused family therapy: why, when and how?
Source: J Eat Disord. 2021 Aug 10;9:97. doi: 10.1186/s40337-021-00451-3 (PMC8353776; doi:10.1186/s40337-021-00451-3)
Supplement: Supplementary file 1 — Additional file 1. [file 40337_2021_451_MOESM1_ESM.pdf]

Step 1: *Genogram & network of important relationships*

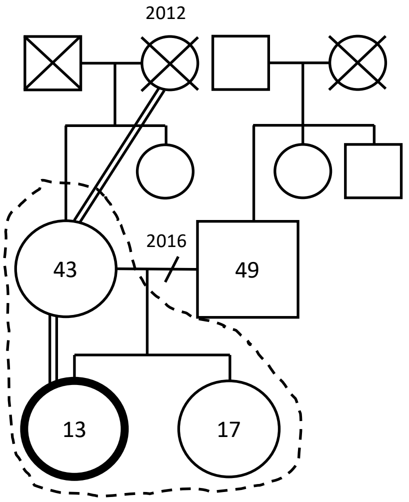

PART ONE: FAMILY CONTEXT

Young person (YP, 13): Lives with mum and sister (17). In second year of high school. Very close with mum.

Mum: Working full time in advertising

Dad: Employed full-time as an actor. Living 45m drive away. See daughters on weekends.

Sister (17): Final year of high school. Spending increasing amounts of time with boyfriend

Grandparents: All born in Spain. Three of four died relatively young. Most recent was maternal grandmother, who was very involved in family life and a great support to mother

PART TWO: DIFFICULTIES IN CONTEXT

Step 2: *Predisposing factors*

- “very active” / “very neat” / “perfectionist” / “high achiever” / “shy”
- Generally quiet, but could have occasional, very large, outbursts as child
- Separation difficulties when starting primary school (~age 5) (no treatment)

Context:

- Both parents quite outgoing and loud
- Sisters both very sociable and made friends easily – ‘always felt like the odd one out’
- Mother always perceived daughter as ‘needy’
  History of OCD on maternal side of the family

Step 3: *Precipitating factors*

- Family “health kick” after mum had breast cancer scare (12 months ago) – increased family exercise, more vegetarian dinners, switch to non-dairy alternatives in the family
- Lonely since moving to high school / struggling to settle into a “group” (age 12)
- YP feels unimportant/unnoticed

Context

- Father perceived friendship difficulties as ‘no big deal’ and something you should ‘get used to’
- Mother very focussed on supporting sister (17) end-of-high-school exams this year
- Maternal guilt over suggesting family ‘health kick’

Step 4: *Presenting concerns:*

- Restriction in food volume and variety past 9-12 months
- Currently vegetarian plus no dairy and no ‘sweets’ (chocolate, cake, desserts)
- ~7 kg weight loss in past 6 months – currently 82%mBMI / heart rate 57
- Secretive exercise (HIIT workouts in bedroom, walking to school (4km) instead of catching the bus)
- Low mood / some scratching of back of hands when distress
- Strong body image concerns and distress regarding weight gain – lots of mirror checking of stomach

Context:

- Dad confused by symptoms and thinks it is ‘just a phase’
- Sister (17) frustrated as trying to study for exams and finding distress at home difficult to manage
- Mum torn between guilt for introducing ‘health kick’ and frustrated because there is a lot going on

Step 5: *Protective factors*

- Family generally supportive
- Sought treatment early
- Likes year 8 teacher and enjoys school
- Has lots of interests/hobbies (band, rugby
- Motivated to ‘feel different’

Context:

- Part of close-knit ex-patriot Spanish community
- Good communication between parents

Step 6: *Perpetuating factors*

- Parents struggle to understand YP rigidity and stubbornness around eating and exercise – invalidating at times despite best intentions
- High expressed emotion at home

Context:

- Family not particularly psychologically minded
- 2 friends in peer group have eating difficulties
- Sister currently trying to lose some weight for a part in the school play

Step 6 (cont.): *Perpetuating patterns*

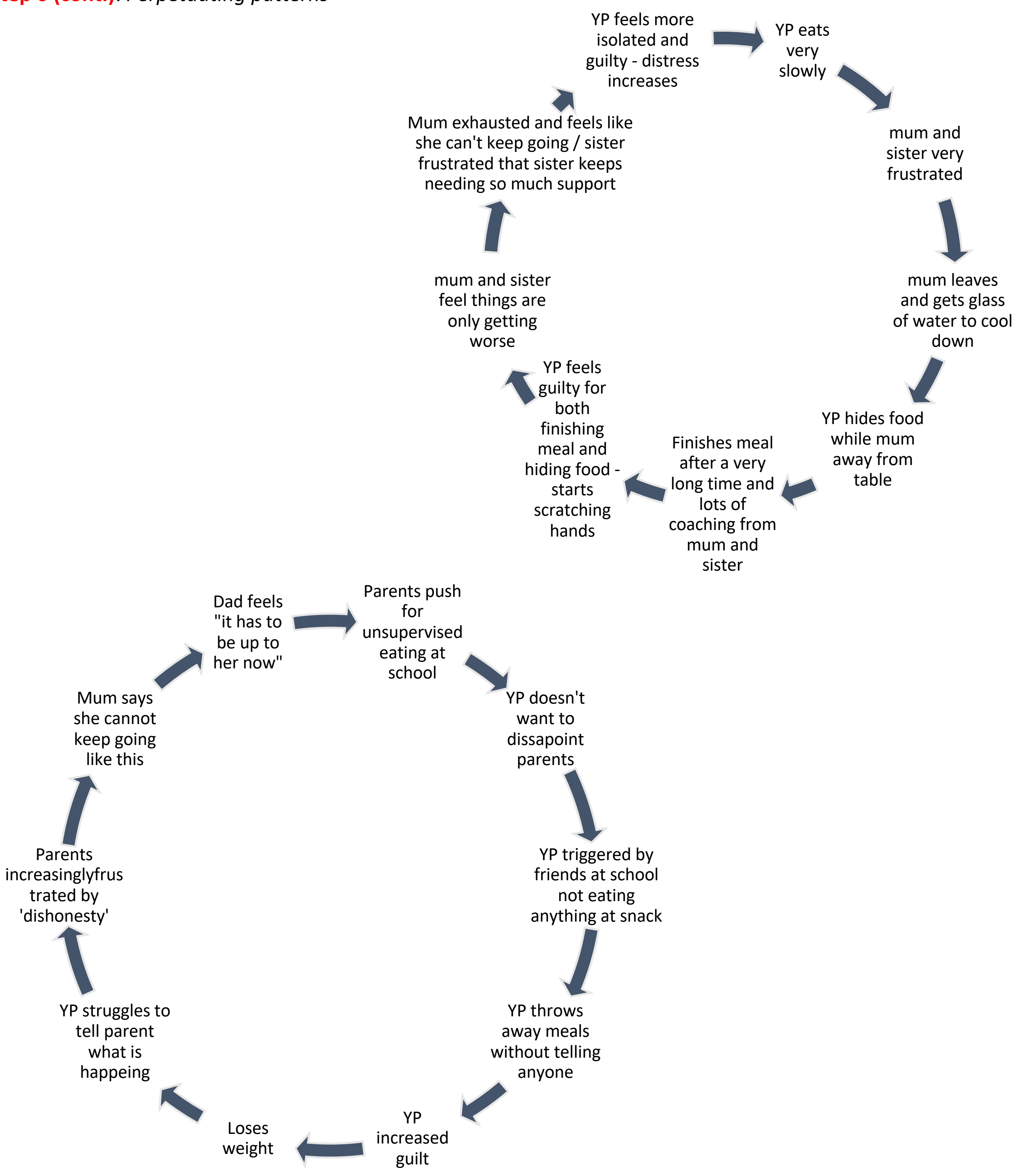

Abbreviations: YP, young person

Step 9: *Plan*

Goals for next review period:

1. Separated sessions until next review
2. Introduce validation and emotion coaching for parents
3. Introduce self-sooth and distress tolerance skills for all family members
4. Support young person to speak about impact of choice/difficulties with independent eating

Review: 1 month
